# Supplementary material for: Predictive value of anthropometric measurements in survival and free walking ability of geriatric hip fractures after surgery
Source: PLoS One. 2024 May 15;19(5):e0303067. doi: 10.1371/journal.pone.0303067 (PMC11095725; doi:10.1371/journal.pone.0303067)
Supplement: Attachment — Submitted filename: Response to Reviewers.docx [file pone.0303067.s002.docx]

**Response Letter**

**Manuscript ID number: PONE-D-23-24968**

**Title: Predictive value of anthropometric measurements in survival and free walking ability of geriatric hip fractures after surgery**

Dear Editors and Reviewers,

Thanks very much for taking the time to review this manuscript. We appreciate all your generous comments and suggestions! Changes to our manuscript have been highlighted by using colored text in the revised manuscript. Please find our response below and my revisions in the re-submitted files.

**Responses to editors**

Thank you for your comments and tips concerning our manuscript. We would respond to reviewers and submit the revised manuscripts according to your prompts. Thank you.

According to the journal requirements, we have edited our manuscript to meet PLOS ONE’s style requirements, and the ORCID would be updated in EM system. Moreover, the duplicated ethics statement has been deleted in our revised manuscript, and the reference to Figure 1 was also added. Lastly, the reference list has also been checked.

**Responses to reviewer 1**

**The article expressed original thoughts with most of the various subsections involved in scientific work given. However, the sample size used for the research was missing including the location of the study otherwise a good article.**

**Response:**

Thank you for your kind comments. Our study included 544 individuals, and this study was conducted at the Emergency Trauma Center of Nanyang Second People’s Hospital. Due to the nature of observational study, we may not control the sample size accurately after following up. Therefore, we set a minimum sample size by calculating the minimum sample size required for multivariable regression. This study included 8 anthropometric measurements variables, and we calculated the sample size by (events per variable) EPV method.

EPV = number of events / number of variables

When the EPV > 10, the multivariable models could be considered being stable. Therefore, the sample size was least 80 for events group [1].

Moreover, according to the similar studies in hip fractures, the sample size of our study (544 individuals) was also satisfactory [2].

More discussion of sample size and location of this study have added to our study (Method and Result part).

[1] Vittinghoff E, McCulloch CE. Relaxing the rule of ten events per variable in logistic and Cox regression. Am J Epidemiol. 2007 Mar 15;165(6):710-8. doi: 10.1093/aje/kwk052. Epub 2006 Dec 20. PMID: 17182981.

[2] Liu M, Chu Q, Yang C, Wang J, Fu M, Zhang Z, Sun G. The paradoxical relation between serum uric acid and outcomes of hip fracture in older patients after surgery: A 1-year follow-up study. Surgery. 2022 Nov;172(5):1576-1583. doi: 10.1016/j.surg.2022.07.008. Epub 2022 Aug 26. PMID: 36031447.

**Responses to reviewer 2**

**The manuscript was well-written however, I have one question regarding anthropometric measurements choices utilised in your research they did not include central obesity measure will this not have affected your outcome and does it mean that all your subjects have no obese individual from the start to finish the observational study.**

**Response:**

Thank you for your valuable advice. We have to admit that we cannot included all central obesity measures in our study. However, we believe some of measurements may be used as central obesity measures, such as waist circumference, thigh circumference, and suprailiac skinfold. Moreover, many studies used waist circumference as an index of central fatness [1]. Therefore, we believe the waist circumference in our study may be considered as a central fatness measure.

[1] Jayedi A, Soltani S, Zargar MS, Khan TA, Shab-Bidar S. Central fatness and risk of all cause mortality: systematic review and dose-response meta-analysis of 72 prospective cohort studies. BMJ. 2020 Sep 23;370:m3324. doi: 10.1136/bmj.m3324. PMID: 32967840; PMCID: PMC7509947.
