# Supplementary material for: Predictive value of anthropometric measurements in survival and free walking ability of geriatric hip fractures after surgery
Source: PLoS One. 2024 May 15;19(5):e0303067. doi: 10.1371/journal.pone.0303067 (PMC11095725; doi:10.1371/journal.pone.0303067)
Supplement: Attachment — Submitted filename: Response to Reviewers.docx [file pone.0303067.s003.docx]

**Response Letter**

**Manuscript ID number: PONE-D-23-24968**

**Title: Predictive value of anthropometric measurements in survival and free walking ability of geriatric hip fractures after surgery**

Dear Editors and Reviewers,

Thanks very much for taking the time to review this manuscript. We appreciate all your generous comments and suggestions! Changes to our manuscript have been highlighted by using colored text in the revised manuscript. Please find our response below and my revisions in the re-submitted files.

**Responses to editors**

Thank you for your comments and tips concerning our manuscript. We would respond to reviewers and submit the revised manuscripts according to your prompts. Thank you.

**Responses to Reviewer 1**

**The author has provided answers to concerns raised: sample size as well as the study location have been satisfactory stated.**

**Response:**

Thank you for your kind comments. I'm glad to hear that the concerns raised regarding the sample size and study location have been satisfactorily addressed.

**Responses to Reviewer 2**

**The author has provided answers to concerns raised: sample size as well as the study location have been satisfactory stated.**

**Response:**

Thank you for recommending the acceptance of the manuscript for publication. We are grateful for your support.

**Responses to Reviewer 3**

**Abstract:**

**The results and conclusion sections of the abstract are rather confusing. It would be beneficial to restructure these sections for better clarity and coherence, ensuring that key findings and implications are presented in a straightforward manner.**

**Response:**

Thank you for your valuable advice. The abstract has been revised as follows:

**[Background:** We aimed to explore the predictive value of anthropometric measurements in survival and free walking ability of geriatric hip fractures after surgery.

**Methods:** Eight common anthropometric measurements, including arm circumference (AC), waist circumference (WC), thigh circumference (TC), calf circumference (CC), biceps skinfold (BS), triceps skinfold (TS), suprailiac skinfold (SIS), and subscapular skinfold (SSS), were included to identify their predictive value in survival and free walking ability of geriatric hip fractures. The results of anthropometric measurements were compared between patients with different outcomes. Cox and logistics models were established to further identify the predictive value of anthropometric measurements.

**Results:** Comparison among groups indicated that individuals with different outcomes may have significantly different anthropometric measurements. In the Cox analyses based on all individuals, all models proved that the patients with higher AC, as well as CC and BS, may have a lower risk of 1-year mortality. Similarly, in the logistics analysis, AC, CC, and BS were proven to have strong predictive ability for 6-month and 1-year mortality in females and overall individuals. However, the predictive value of the eight common anthropometric measurements in free walking ability is not significant.

**Conclusion:** AC, CC, and BS may have strong predictive ability for 6-month and 1-year mortality in all individuals and females.]

**Introduction:**

**The language used in the introduction is problematic, with notable issues of redundancy and unclear phrasing. A thorough revision to improve the language and eliminate repetitive statements is necessary to enhance the quality and readability of this section.**

**Response:**

Thank you for your valuable advice. The introduction part has been revised thoroughly with the help of native English speakers to enhance the quality and readability.

This part has been revised as follows:

[Hip fractures, occurring in the proximal femur, are commonly considered one of the most dangerous fractures in elder adults [1]. These fractures are primarily caused by low-energy trauma, often resulting from falls, while the root causes are age-related factors such as impaired balance, muscle weakness, and decreased bone mineral density [2]. Geriatric hip fractures are associated with high morbidity and mortality rates [3], primarily due to pre-existing comorbidities, surgical complications, and postoperative immobility [4]. Common complications include surgical site infections,

urinary tract infections, venous thromboembolism, pressure ulcers, pneumonia, and delirium.

Nutrition status is a major risk factor for the outcomes of hip fractures and significantly impacts the rehabilitation of geriatric hip fractures [5]. Adequate nutrition is essential for bone regeneration, tissue repair, immune function, and wound healing, all of which may reduce the incidence of complications and improve the prognosis of geriatric hip fractures [6]. However, malnutrition as a common concern in older adults may contribute to poor healing, increased complications, prolonged recovery, low survival, and high disability following a hip fracture [7].

Assessing and addressing the nutritional needs in older adults with hip fractures is vital to improving their outcomes [5]. Anthropometric measurements, such as arm circumference, waist circumference, and others, can reflect an individual's nutritional status [8]. Poor anthropometric measurements are often associated with malnutrition, which can negatively impact the healing process and functional recovery in geriatric hip fracture patients. These anthropometric measurements provide valuable information about body composition and can assist healthcare professionals in assessing the nutritional needs of older adults with hip fractures. In this study, eight common anthropometric measurements, including arm circumference (AC), waist circumference (WC), thigh circumference (TC), calf circumference (CC), biceps skinfold (BS), triceps skinfold (TS), suprailiac skinfold (SIS), and subscapular skinfold (SSS), were included to identify their predictive value in survival and free walking ability of geriatric hip fractures.]

**Methods:**

**The study exhibits a considerable loss to follow-up (33%, including lost data), which could introduce significant bias into the conclusions. It's crucial to address this issue and discuss its potential impact on the study's validity.**

**Response:**

Thank you for your valuable advice. We noted that the high rate of loss to follow-up may cause bias in our study. However, we have tried our best to control the sample size in this study. Assuming that loss to follow-up is a random event, if the sample size is sufficient, the patients who are not lost to follow-up can adequately represent all patients. We believe that the bias introduced by loss to follow-up should also be controllable. Similarly, previous studies have also reported similar rates of loss to follow-up (PMID: 36510169, 36127624). More discussion about this issue has been added to the limitation part in our revised manuscript.

The following part has been added to our revised manuscript:

[Firstly, as an observational study, this study had data loss and loss to follow-up, which may introduce bias into our findings. However, we have tried our best to control the sample size in this study. Assuming that loss to follow-up is a random event, if the sample size is sufficient, the patients who are not lost to follow-up can adequately represent all patients. We believe that the bias introduced by loss to follow-up should also be controllable.]

**The decision to separate analyses by sex to reduce gender bias is noted. However, it seems that incorporating these variables into regression models could further minimize bias. Could you please explain the rationale behind not using such methods in your analysis?**

**Response:**

Thank you for your valuable comments. We noticed that we forgot to state the specific variables for each model in the manuscript. In fact, in the Cox and logistic regression models, we have included the variables that showed significant differences in the univariate analysis. The included variables are age, sex (in models for all individuals only), BMI, and fracture type (femur neck and intertrochanteric). This variable selection approach is also commonly used in most literature, as it allows for the inclusion of relevant variables while ensuring model stability.

We decided to conduct a gender subgroup analysis because we realized that differences in body measurements between different genders could lead to bias. Discrepancies in cutoff points, averages, and other measurements could potentially impact the outcomes. As a result, we chose to conduct a gender subgroup analysis to better understand how these differences may influence the results.

More description of the variables in multivariable models has been added to our revised manuscript.

The following part has been added to our revised manuscript:

[Age, sex (in models for all individuals only), BMI, and fracture type were included in the Cox models 1 and 2 as co-variables.

Age, sex (in models for all individuals only), BMI, and fracture type were included in the multivariable logistics models 1 and 2 as co-variables.]

**Results:**

**The tables would benefit from including the number of patients involved in each specific analysis. This addition would greatly assist in understanding the scope and applicability of the findings.**

**Response:**

Thank you for your valuable advice. Added accordingly. Please see the tables in our revised manuscript.

**Determining and specifying a cut-off value for each measurement under study would significantly enhance the interpretative value of the results.**

**Response:**

Thank you for your valuable advice. We added an independent part for ROC curves to our study, and the detailed results of ROC analysis, including AUROC, 95%CI, potential cutoff values, and p values, have been added to our revised manuscript (Table 4 and supplementary table 11). Related descriptions have also been added to our revised manuscript.

The following parts have been added to our revised manuscript:

[Similar to Cox models, the logistics modes 1 and 2 were also established by adjusting for all types or single type of anthropometric measurements, respectively. ROC curves were established to evaluate the predictive values and to determine the potential cut-off values for each measurement. To avoid the bias caused by sex, the analyses were also performed in males and females separately.]

**[Predictive values of anthropometric measurements**

ROC curves were established to determine the predictive values and the potential cutoff values of anthropometric measurements for mortality and free walking ability. The multivariable analysis indicated the predictive ability of AC, CC, and BS, so we summarized their detailed results of ROC curves in Table 4. The detailed results of ROC analyses for all anthropometric measurements, including AUC, 95%CI, potential cutoff values, and p values, were summarized in Supplementary table 11. Similarly, the AC, CC, and BS showed significant predictive values for 1-year mortality in all individuals and females, while no significant results in males. For 1-year free walking ability, AC was proven to have significant predictive ability in all individuals and females, and CC was only proven in all individuals, while no significant results of BS were proven. The potential cutoff values of each anthropometric measurement have been summarized in Table 4 and supplementary table 11.

Table 4: Results of ROC analysis for AC, CC, and BS.

| All individuals | | | | | | |
| --- | --- | --- | --- | --- | --- | --- |
|  | 1-year mortality | | | 1-year free walking ability | | |
|  | AUC(95% CI) | Cutoff values | p | AUC(95% CI) | Cutoff values | p |
| AC | 0.595 (0.529, 0.661) | 21.88 | 0.002 | 0.568 (0.513, 0.623) | 28.155 | 0.008 |
| CC | 0.597 (0.528, 0.667) | 29.61 | 0.001 | 0.571 (0.514, 0.627) | 29.625 | 0.007 |
| BS | 0.591 (0.526, 0.656) | 13.005 | 0.003 | 0.523 (0.465, 0.581) | 13.035 | 0.791 |
| Males | | | | | | |
|  | 1-year mortality | | | 1-year free walking ability | | |
|  | AUC(95% CI) | Cutoff values | p | AUC(95% CI) | Cutoff values | p |
| AC | 0.556 (0.426, 0.687) | 23.25 | 0.156 | 0.556 (0.451, 0.661) | 26.295 | 0.87 |
| CC | 0.581 (0.459, 0.702) | 29.59 | 0.074 | 0.664 (0.564, 0.763) | 29.65 | 0.001 |
| BS | 0.600 (0.488, 0.712) | 13.01 | 0.964 | 0.539 (0.440, 0.639) | 13.165 | 0.786 |
| Females | | | | | | |
|  | 1-year mortality | | | 1-year free walking ability | | |
|  | AUC(95% CI) | Cutoff values | p | AUC(95% CI) | Cutoff values | p |
| AC | 0.617 (0.542, 0.691) | 28.245 | 0.002 | 0.575 (0.510, 0.640) | 28.31 | 0.015 |
| CC | 0.608 (0.523, 0.694) | 29.395 | 0.004 | 0.524 (0.455, 0.594) | 23.19 | 0.76 |
| BS | 0.589 (0.508, 0.670) | 12.98 | 0.014 | 0.517 (0.445, 0.589) | 12.98 | 0.686 |

Note: AC: arm circumference, CC: calf circumference, BS: biceps skinfold.]

[Supplementary table 11: Detailed information of ROC curves; Supplementary table 12: Raw data of our study.]

**Consideration should be given to including more variables in your models, such as sex, height, weight, BMI, age, etc., to provide a more comprehensive analysis.**

**Response:**

Thank you for your valuable advice. We noticed that we forgot to state the specific variables for each model in the manuscript. In fact, in the Cox and logistic regression models, we have included the variables that showed significant differences in the univariate analysis. The included variables are age, sex (in models for all individuals only), BMI, and fracture type (femur neck and intertrochanteric). This variable selection approach is also commonly used in most literature, as it allows for the inclusion of relevant variables while ensuring model stability. More description of the variables in multivariable models has been added to our revised manuscript.

The following part has been added to our revised manuscript:

[Age, sex (in models for all individuals only), BMI, and fracture type were included in the Cox models 1 and 2 as co-variables.

Age, sex (in models for all individuals only), BMI, and fracture type were included in the multivariable logistics models 1 and 2 as co-variables.]
