# Supplementary material for: Predictive value of anthropometric measurements in survival and free walking ability of geriatric hip fractures after surgery
Source: PLoS One. 2024 May 15;19(5):e0303067. doi: 10.1371/journal.pone.0303067 (PMC11095725; doi:10.1371/journal.pone.0303067)
Supplement: Attachment — Submitted filename: Response to Reviewers.docx [file pone.0303067.s004.docx]

**Response Letter**

**Manuscript ID number: PONE-D-23-24968**

**Title: Predictive value of anthropometric measurements in survival and free walking ability of geriatric hip fractures after surgery**

Dear Editors and Reviewers,

Thanks very much for taking the time to review this manuscript. We appreciate all your generous comments and suggestions! Changes to our manuscript have been highlighted by using colored text in the revised manuscript. Please find our response below and my revisions in the re-submitted files.

**Responses to editors**

Thank you for your comments and tips concerning our manuscript. We would respond to reviewers and submit the revised manuscripts according to your prompts. Thank you.

**Responses to Reviewer 4**

**a. Line 93; Please further elaborate on how CCI is calculated and interpreted.**

**Response:**

Thank you for your valuable advice. More description on CCI has been added to our revised manuscript.

The following part has been added to our revised manuscript:

**[**CCI is a widely used tool in medical research and clinical practice to quantify the burden of comorbidities in patients. The index takes into account 19 different conditions, such as heart disease, diabetes, and cancer, and assigns a score ranging from 1 to 6 to each condition based on its severity and impact on mortality [9]. The items and their weights were summarized in Supplementary table 1.

9.	Charlson ME, Carrozzino D, Guidi J, Patierno C. Charlson Comorbidity Index: A Critical Review of Clinimetric Properties. Psychother Psychosom. 2022;91(1):8-35. Epub 20220106. doi: 10.1159/000521288.]

**b. Please describe a summary of participants’ characteristics (at least their age and gender) in the first paragraph of the results section.**

**Response:**

Thank you for your valuable advice. Added accordingly.

The following part has been added to our revised manuscript:

[The mean age of the study population was 72.38 ± 10.47 years, and the mean BMI was 21.77 ± 4.27 kg/m^2^. More female participants (374, 68.75%) were included in this study than males (170, 31.25%).]

**c. Table 1 may be somewhat misleading, and I recommend omitting it. Since males and females possess distinct anthropometric characteristics, aggregating them to assess the association between anthropometric measures and outcomes is not advisable. Tables 2 and 3 effectively convey the relationship between these measures and the outcome. I recommend a similar approach and omitting the aggregated section of Table 4, Figure 2, and Figure 3.**

**Response:**

Thank you for your valuable advice. These tables and figures have been revised accordingly. The updated Tables 1, 2, and 3 were the previous Tables 2, 3, and 4. The previous table 1 has been added to supplementary tables.

**d. Please add a conclusion section at the end of the discussion section.**

**Response:**

Thank you for your valuable comments. Added accordingly.

The following part has been added to our revised manuscript:

[Conclusion

The predictive value of the eight common anthropometric measurements in elderly male patients after hip fracture surgery may not be significant in terms of mortality and rate of free walking. Additionally, their predictive role in free walking ability is also not significant in the both overall population and females. However, in all individuals and females, AC, CC, and BS have strong predictive ability for 6-month and 1-year mortality.]
